# Prospero-related homeobox protein 1 (Prox1) enhances myelin sheath regeneration in injured sciatic nerve

**DOI:** 10.1016/j.ibneur.2025.09.011

**Published:** 2025-09-24

**Authors:** Linlin Jie, Xuening Jing, Jie Liu, Jun Ren, Xiaoni Kong, Jun Wu, Fanwei Meng

**Affiliations:** Department of Anatomy and Physiology, Shandong College of Traditional Chinese Medicine, Yantai, Shandong 264199, China

**Keywords:** Sciatic nerve injury, Myelin sheath injury, Myelin sheath regeneration, Prox1, MBP, Schwann Cells, Peripheral Nerve Injury

## Abstract

**Background:**

Peripheral nerve injury (PNI), such as sciatic nerve injury (SNI), often results in poor functional recovery due to myelin damage, which critically impairs nerve regeneration. Prox1, a homeobox transcription factor known to influence neurogenesis, has not been studied in peripheral myelin regeneration.

**Objective:**

To investigate morphological changes in myelin sheaths and the expression of Prox1 and MBP proteins during sciatic nerve repair, and to determine the role of Prox1 in nerve regeneration.

**Methods:**

Mice were divided into seven groups (n = 8): one control and six experimental groups based on sciatic nerve collection at Days 3, 4, 5, 6, 7, and 14 post-injury. H&E staining, electron microscopy, Luxol Fast Blue staining, Western Blot, and immunohistochemistry were used to assess myelin morphology and Prox1/MBP expression. Immunofluorescence analyzed colocalization of Prox1 and MBP. Prox1 was overexpressed in Schwann cells via plasmid transfection, and MBP levels were measured by Western blot.

**Results:**

Control group myelin sheaths showed normal structure, while Day 7 nerves displayed disorganized sheaths with vacuolation and axonal spacing. By Day 14, myelin structure largely recovered. MBP levels decreased from Day 3, reached a minimum at Day 7, and increased significantly by Day 14. Prox1 expression rose on Day 3, peaked on Day 7, and declined by Day 14. Prox1 and MBP colocalized in injured nerves, and Prox1 overexpression significantly increased MBP levels in Schwann cells.

**Conclusion:**

Prox1 protein level is upregulated in injured sciatic nerve, and Prox1 overexpression promotes the increase of MBP protein level, which positively correlates to myelin regeneration in morphology. This strongly suggests that Prox1 promotes myelin regeneration of injured peripheral nerves.

## 1 Introduction

Peripheral nerve injury (PNI) is a common clinical condition characterized by poor prognosis, slow recovery, long-term neuropathic pain, and permanent impairment of motor function after illness, which caused psychological distress to patients, imposed a significant burden on society, and making PNI a fundamental challenge in clinical medicine (Zhang et al., 2021).

Sciatic nerve injury (SNI) is a common type of peripheral nerve damage and has been commonly used as a model in peripheral nerve injuries related study (Wang et al., 2024, Zhao et al., 2022). SNI is one of the most common types of PNI. In pathology, PNI primarily manifests as neuropathy, axonal rupture, nerve transection, and Wallerian degeneration of distal nerve fibers. Myelin damage observed in PNI significantly affects the regeneration and functional recovery of peripheral nerves (Yuan et al., 2022). Myelin is an outside membrane wrapping the axons of nerve cells, and abnormalities in myelin can lead to various diseases (Wright, 2023, Florian et al., 2021, Noorani et al., 2019). Myelin regeneration has been the key point of treating PNI (Yuan et al., 2022), with the myelin sheath regeneration positively correlates with the expression level of myelin basic protein (MBP) (Liu et al., 2019). The mechanisms, timing, and regulatory factors involved in myelin regeneration have remained challenging obstacles to overcome.

Prospero-related homeobox protein 1 (Prox1) is a crucial member of the homeobox transcription factor family, playing a significant role in the development of tissues and organs such as the nerves, liver, heart, and lymphatic system during vertebrate embryonic development. Prox1 also plays a crucial role in neurogenesis and the differentiation of neurons (Miyoshi et al., 2015, Elkouris et al., 2011), and has been found to promote the differentiation of oligodendrocyte precursor cells into oligodendrocytes in the hippocampal DG area and the subventricular zone (Gomez-Sanchez et al., 2017). However, there are no reports on whether Prox1 promotes myelin sheath regeneration in the peripheral nervous system.

In our previous study, Prox1 protein expression was found to change significantly at 7 days and 14 days following SNI (Meng et al., 2020). While the injury and regeneration of the myelin sheath have been extensively documented, the spontaneous repair timeline of myelin sheaths in injured sciatic nerves remains poorly understood, particularly regarding the role of Prox1 in myelin sheath repair of injured sciatic nerves. To address this gap, we investigated myelin sheath damage and regeneration, along with Prox1 expression, at various time points post-injury. This study aims to clarify the mechanisms of myelin regeneration and functional recovery following SNI, with a focus on Prox1's role in mediating these processes, providing a theoretical foundation for clinical treatment of injured sciatic nerves

## 2 Materials and methods

### 2.1 Key reagents and equipment

Rabbit anti-Myelin basic protein/MBP Polyclonal antibody (Proteintech, USA, catalog # 10458–1-AP); Mouse anti-MBP tag Monoclonal antibody (Proteintech, USA, catalog # 66003–1-Ig); Anti-PROX1 Antibody (Boster Bio, China, catalog # A01985–1); DAB Chromogenic Substrate Reagent Kit (Blue) (Boster Bio, China, catalog # AR1025); Rabbit Anti-GAPDH (Jiangsu KeyGEN BioTECH, China, catalog # KGC6102–1); Luxol Fast Blue Myelin Stain Kit (Solarbio, China, G3240); KeygenMAX 3000 transfection reagent (Jiangsu KeyGEN BioTECH, China, catalog # KGA9705–1); PrimeScript™ RT Master Mix (TaKaRa, Japan, catalog # RR036B); Olympus Inverted Microscope (OLYMPUS, Japan, catalog # IX51); ABI Stepone real-time PCR (Thermo Fisher, USA); Trans-Blot® Turbo™ Transfer System (BIO-RAD, USA, catalog #1704150).

### 2.2 Animal model and specimen preparation

This animal study was approved by the Ethics Committee of the Shandong College of Traditional Chinese Medicine, China and the Approval Number is 2022–02. All the animal treatments followed the ethical committee's guidelines strictly. All animals were housed in a specific pathogen-free animal facility at Shandong College of Traditional Chinese Medicine, at the constant temperature of 24°C, humidity at 60 %, and a normal light cycle. Fifty-six male adult Kunming mice, weighing between 18 and 26 g, were randomly divided into seven groups (n = 8 each group): control group, Day 3, Day 4, Day 5, Day 6, Day 7, and Day 14 experimental group. The control group underwent no treatment, while the sciatic nerve of the other six groups were injured by referring to the protocol from Meng et al (Meng et al., 2020). In brief: mice were anesthetized intraperitoneally. Make a longitudinal incision along the midline of the posterior thigh, then separate the biceps femoris and semimembranosus muscles to expose the sciatic nerve. Vascular forceps were used to alternately clamp and release a 1 cm segment of the sciatic nerve, with each compression lasting 10 s and repeated three times. Non-invasive clamps (using 0-gauge surgical suture) were applied to mark the upper and lower ends of the sciatic nerve. Post-surgery, the skin incision was closed using 7-gauge surgical suture. Animals were euthanized for sciatic nerve sample collection at 3 days-, 4 days-, 5 days, 6 days, 7 days, and 14 days-post injury by intraperitoneal anesthesia, cervical dislocation, followed by the isolation of the sciatic nerve.

### 2.3 Hematoxylin and eosin (H&E) staining

The collected sciatic nerve was fixed in 4 % formaldehyde and embedded in paraffin, which was sectioned to 5 µm section and grilled for 30 min.The H&E staining on sections was processed by referring to the previous publication (Li et al., 2018, Cardiff et al., 2014). In brief, the 4 µm section slides were deparaffinized and rehydrated by sequential immersion in xylene (10 min), ethanol solutions (100 %, 95 %, 90 %, 80 %, 70 %; 5 min each), and distilled water (2–5 min). The slides were then stained with hematoxylin for 3 min, destained in 1 % acid ethanol, rinsed in tap water, then stained with eosin for 1 min. They were dehydrated through ethanol (70 %, 95 %, 100 %; 5 min each), xylene (5 min each), and mounted with Neutral Balsam. Allow the slides to dry before examining under a light microscope.

### 2.4 Sample preparation for Electron Microscopy (EM)

On the collection day, mice were anesthetized with 4 % chloral hydrate (10 ml/kg), and sciatic nerves were dissected, cut into 1 mm³ cubes, and fixed overnight in 2.5 % glutaraldehyde at 4°C. The next day, the samples were fixed in 1 % osmium tetroxide for 3 h, dehydrated through ethanol and acetone gradients, and embedded in resin at 60°C for 48 h. Ultrathin sections (60–80 nm) were cut, stained with 2 % uranyl acetate and lead citrate for 10 min, and observed under a transmission electron microscope (HITACHI).

### 2.5 Immunohistochemistry (IHC)

The IHC was processed by referring to the paper (Xiang et al., 2005). Place 5 µm paraffin-embedded tissue sections on slides and dry for 30 min. Heat at 60°C for 30 min to ensure tissue adherence. Dewax in xylene (2 ×5 min) and rehydrate through ethanol (100 %, 95 %, 70 %; 5 min each) washes, followed by a distilled water rinse. Perform antigen retrieval by incubating in 0.01 M citrate buffer at 121°C for 15 min, then cool and rinse in PBS. Incubate with 3 % hydrogen peroxide for 20 min, block with 10 % sheep serum, and incubate overnight at 4°C with primary antibodies (1:100, rabbit anti-mouse MBP, or rabbit anti-mouse Prox1). The next day, incubate with HRP-labeled secondary antibody at 37°C for 30 min and stain with DAB. Images were analyzed using Image J/Fiji for optical density and Image-Pro Plus for quantifying MBP or Prox1 positive cells.

### 2.6 Immunofluorescence (IF)

Paraffin-embedded 5 µm sections were deparaffinized in xylene and alcohol, then antigen-retrieved in 0.01 M citrate buffer at 121°C for 15 min. After 20 min in 3 % hydrogen peroxide, the sections were blocked with 10 % goat serum for 1 h and incubated overnight with rabbit anti-mouse MBP (1:100). The next day, sections were incubated with Cy3-labeled goat anti-rabbit secondary antibody (1:300) for 40 min, washed, and mounted with anti-fading medium for fluorescence microscopy. For double immunofluorescence, primary antibodies (rabbit anti-mouse Prox1 and mouse anti-mouse MBP) and secondary antibodies (Cy3-goat anti-rabbit and Alexa Fluor-488-donkey anti-mouse) were used.

### 2.7 Luxol Fast Blue staining

After dissection, the sciatic nerve was fixed in 4 % formaldehyde, embedded in paraffin, and sectioned into 6 µm slices. The sections were deparaffinized in xylene, anhydrous ethanol, and 95 % ethanol, then stained overnight in Luxol Fast Blue solution. The next day, excess stain was washed off with 95 % ethanol and distilled water. Sections were differentiated briefly in 70 % ethanol, then re-stained with Eriochrome solution, dehydrated in 95 % and 100 % ethanol for 2 min, and cleared in xylene for 5–10 min. Coverslip was sealed and examined under a microscope.

### 2.8 Plasmid or siRNA transfection on seeded cells

To investigate PROX1 knockdown effects on MBP expression, siRNA was used to silence PROX1 in mouse Schwann cells cultured at 37°C with 5 % CO2. The experimental groups included: Schwann cells, Schwann cells + siRNA NC, and Schwann cells + siRNA-Prox1. Cells were seeded to 70–80 % confluency, then transfected with 2.5 μL of 20 μM siRNA and 5 μL of KeygenMAX reagent, followed by incubation for 6 h. After replacing the medium, cells were cultured for 48 h. To study the level change of MBP proteins, we also overexpressed PRXO1 with pcDNA-Prox1 plasmid transfection. Schwann cells were transfected with either pcDNA-empty vector or pcDNA-Prox1 plasmid using a mixture of 2 µg plasmid and 5 μL KeygenMAX reagent, with incubation for 48 h prior to collection.

### 2.9 RNA extraction

Total RNA was extracted from mouse Schwann cells using TRIzol. Cells were harvested, centrifuged, resuspended in TRIzol, and vortexed. After adding chloroform and centrifuging, the aqueous phase was transferred to a new tube, and RNA was precipitated with isopropanol. After centrifugation, the RNA pellet was washed with 70 % ethanol, air-dried, and dissolved in RNase-free water. RNA concentration and purity were measured using a Nanodrop.

### 2.10 RT-qPCR

Total RNA was extracted and reverse transcribed into cDNA using a TaKaRa kit. Quantitative PCR (qPCR) was performed with SYBR Green Supermix on a thermal cycler: 95°C for 5 min, then 40 cycles of 95°C for 15 s, 60°C for 20 s, and 72°C for 40 s, followed by 95°C for 15 s, 60°C for 1 min, and 95°C for 15 s. The primers used were: PROX1 (Forward: TTCAGCCCGAAAAGAACAGAAG, Reverse: GTGCTGTCATAGACCTGGTAGA); GAPDH (Forward: AAGGTCGGTGTGAACGGATT, Reverse: TGAGTGGAGTCATACTGGAACAT). PROX1 expression was normalized to GAPDH using the ΔΔCt method.

### 2.11 Western Blot

After 48 h of transfection, cells were digested with Trypsin and collected. After centrifugation, PMSF and lysis buffer were added and incubated at 4°C for 15 min. The supernatant was collected, and protein concentration measured by BCA assay. Samples were normalized, mixed with SDS loading buffer, heated, and loaded onto a Tris-Glycine gel. The gel was run, proteins transferred to a PVDF membrane, blocked, and incubated with primary antibodies overnight. After washing, the membrane was incubated with HRP-conjugated secondary antibody, washed, and imaged using HRP substrate on the ChemiDoc MP Imaging System.

### 2.12 Statistical Analysis

IPP 5.1 imaging software was used to analyze MBP and GAPDH protein bands and fluorescent images of MBP and Prox1 positive cells. Data was processed and analyzed with GraphPad Prism 8 software. Quantitative data were expressed as mean ± standard deviation (SD). Statistical significance was determined as P < 0.05.

## 3 Results

### 3.1 Pathological changes of myelin sheaths in injured sciatic nerves under EM

**Fig. 1:**
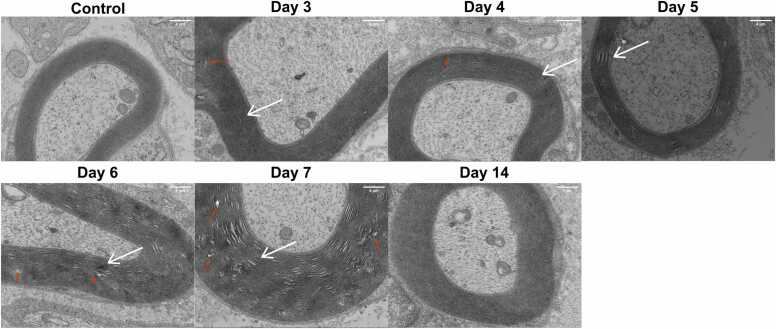
Morphological changes of sciatic nerve myelin sheaths post injury in different groups under EM (×10000). The white arrows: myelin infolding and lamellar separation; red arrows: myelin vacuolation.

EM images (Fig. 1) show that in the control group, the sciatic nerve myelin sheath is organized into dense, concentric lamellae with alternating light and dark bands. In contrast, significant myelin abnormalities, including folding, disarray, vacuoles, and demyelination, first appear on Day 3 and are most pronounced by Day 7. By Day 14, most myelin structures had largely recovered, with a return to a more organized state. These findings highlight the dynamic changes in myelin following injury and the potential for recovery during peripheral nerve regeneration.

### 3.2 Pathological changes of myelin sheaths in injured sciatic nerves of H&E staining

**Fig. 2:**
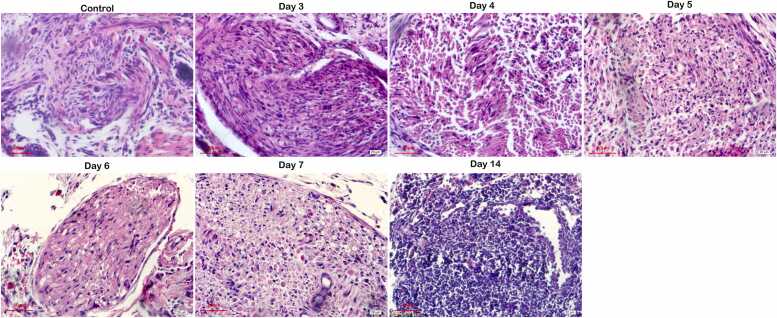
H&E staining of myelin sheaths in injured sciatic nerves on Days 3, 4, 5, 6, 7, and 14 post-injury.

Cell nuclei appear blue and cytoplasm/pink structures in H&E-staining. H&E staining of mouse sciatic nerves (Fig. 2) showed that in the control group, axons were well-organized, with uniform morphology and intact myelin sheaths. Axonal damage was first observed on Day 3 and 4, characterized by vacuoles and increased axonal spacing. By Day 5 and 6, axonal spacing continued to increase, with further disorganization. On Day 7, myelin sheaths were noticeably sparse, disordered, and reduced in quantity. However, by Day 14, axons were more orderly and uniform in size, with increased peripheral myelin fibers and reduced vacuole-like degeneration, axonal swelling, and shrinkage. These findings correlate with electron microscopy observations of myelin changes in injured sciatic nerves.

### 3.3 Pathological changes in myelin sheaths of injured sciatic nerves revealed by luxol fast blue staining

**Fig. 3:**
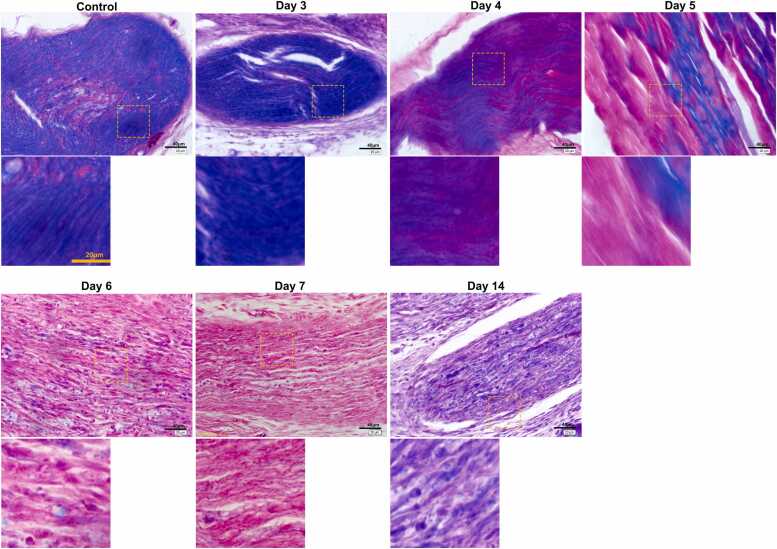
Luxol fast blue staining on myelin sheaths of injured sciatic nerves of control sciatic nerves and injured sciatic nerves on Day 3, 4, 5, 6, 7 and 14 post-injuries.

Luxol fast blue staining (Fig. 3) showed myelin in blue, with a lighter background. In the control group, myelin appeared intact and uniformly blue, with no signs of demyelination. On Day 3 post-injury, significant blue staining indicated some myelin preservation, though this diminished by Day 5, suggesting loss of myelin integrity. By Day 7, the sample primarily exhibited red staining with minimal blue, indicating extensive demyelination. However, on Day 14, blue-stained areas significantly increased, with clear myelin sheath structures, indicating notable recovery and restoration of myelin following injury.

### 3.4 Changes in MBP protein levels in myelin sheaths of injured sciatic nerves

**Fig. 4:**
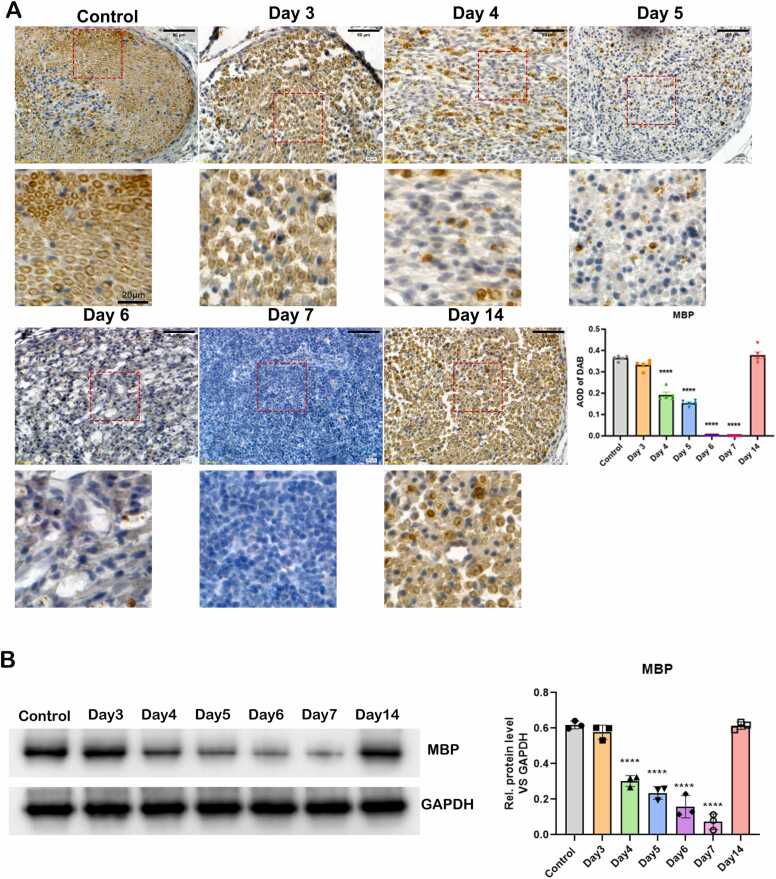
(A) Changes in MBP protein levels in myelin sheaths of control sciatic nerves and injured sciatic nerves on Day 3, 4, 5, 6, 7 and 14 post-injuries. MBP proteins were stained with IHC (×200) and conjugated to DAB. The MBP protein expression was also quantified and displayed in the top right panel. P value was normalized to control group, **** P < 0.0001, n = 5, One-Way ANOVA analysis. (B) WB and quantitative analysis of of MBP protein in myelin sheaths of control sciatic nerves and injured sciatic nerves on Day 3, 4, 5, 6, 7 and 14 post-injuries. **** P < 0.0001, n = 3, One-Way ANOVA analysis.

MBP-positive signals, appearing as brownish-yellow, circular or oval structures in the myelin sheath, were observed in the control group by IHC (Fig. 4 A, images). Quantitative analysis revealed a progressive decrease in MBP levels from Days 3–5 post-injury, with the lowest expression on Day 7. Compared to the control group, MBP levels were significantly reduced on Days 4, 5, 6, and 7 (P < 0.0001, One-Way ANOVA, n = 5). However, by Day 14, MBP expression significantly increased, approaching control levels, indicating notable recovery of myelin integrity (Fig. 4 A, quantification). Consistently, Western blot analysis showed that MBP expression was highest in the normal group and progressively decreased, reaching its lowest level at day 7 (Fig. 4B).

### 3.5 MBP proteins colocalized to Prox1 proteins in injured sciatic nerve

**Fig. 5:**
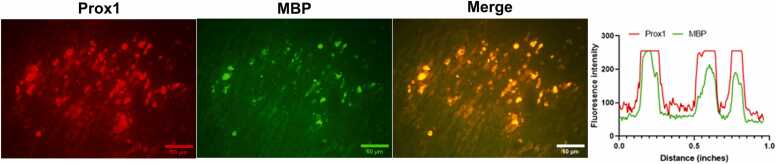
Double IF staining of Prox1 (red) and MBP (green) with IF (×200) and their intensity profile obtained using ImageJ software, along an ideal straight line (white).

Double IF staining revealed significant colocalization of myelin basic protein (MBP) and Prox1 in injured sciatic nerves, with both proteins exhibiting a rounded morphology (Fig. 5). This suggests a potential interaction between MBP and Prox1 during the injury response, indicating that they may play complementary roles in myelin maintenance and repair following PNI. Further studies are needed to explore the functional implications of this interaction in nerve regeneration and myelin dynamics.

### 3.6 Expression level change of Prox1 proteins post sciatic nerve injury

**Fig. 6:**
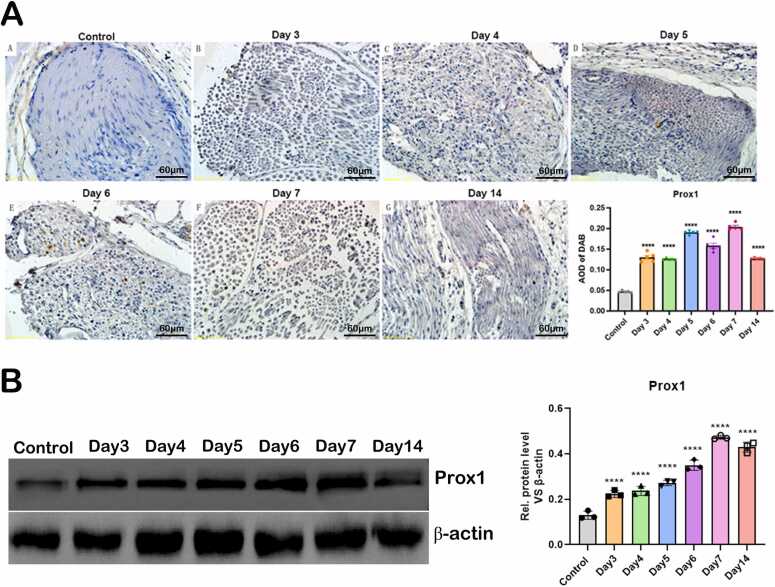
(A) IHC images of Prox1 proteins in sciatic nerve tissue of control sciatic nerves and injured sciatic nerves on Day 3, 4, 5, 6, 7 and 14 post-injuries (×200) and the quantification of Prox1 proteins in top right panel (P value was normalized to control group, **** P < 0.0001, n = 5, One-Way ANOVA analysis was used). (B) Western blot and quantitative analysis of Prox1 protein expression in sciatic nerve tissue from control nerves and injured nerves at days 3, 4, 5, 6, 7, and 14 post-injury (**** P < 0.0001, n = 3, One-Way ANOVA analysis was used).

IHC analysis revealed that Prox1 signals in the control group displayed a brownish-yellow, punctate pattern on the myelin sheath (Fig. 6 A). In contrast, Prox1 expression was significantly elevated in the experimental groups on Days 3, 4, 6, 7, and 14 post-injuries, with darker staining observed in all cases (P < 0.0001 for all comparisons, n = 5, One-Way ANOVA). This suggests an upregulation of Prox1 in response to nerve injury. Notably, Prox1 levels peaked on Day 7, highlighting its critical role in the early repair process. However, Prox1 expression declined by Day 14, indicating a reduced role as recovery progresses. These findings underscore the dynamic regulation of Prox1 during nerve repair and its significance in the early injury response. Furthermore, we verified the expression of Prox1 by Western blot (Fig. 6B). The results showed that Prox1 expression was lowest in the normal group and gradually increased, reaching the highest level at day 7.

### 3.7 PROX1 overexpression led to an increase in MBP protein levels

**Fig. 7:**
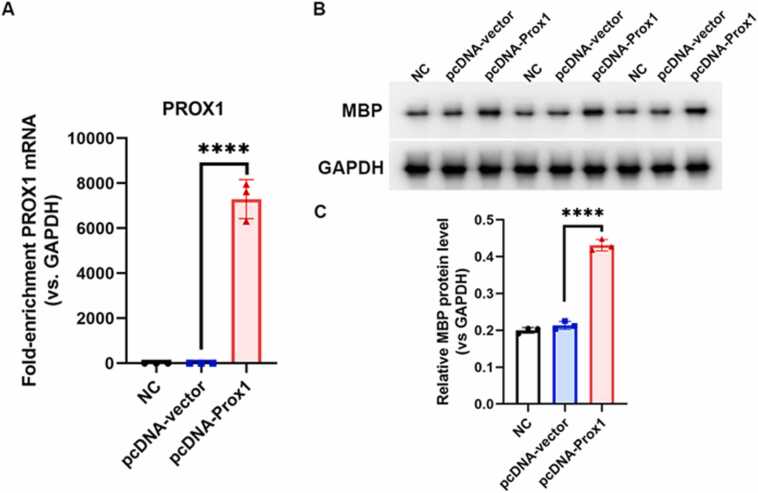
MBP protein level was significantly increased by PROX1 overexpression. (A) qPCR quantification of PROX1 overexpression, (B) WB images of MBP and GAPDH protein from cells transfected with NC, pcDNA-vector or pcDNA-prox1, (C) the quantification of (B). (**** P < 0.0001, n = 3, One-Way ANOVA analysis was used).

Building on previous IHC results for Prox1, double IF staining of Prox1 and MBP, and data from our group (Meng et al., 2020), we found that Prox1 expression peaked at 7 days post-injury, coinciding with the peak of myelin damage. In healthy control mice, Prox1 is expressed at low levels, but it is upregulated in response to injury or during developmental stages. To investigate the impact of Prox1 on myelin regeneration and MBP expression, we overexpressed Prox1 in mouse Schwann cells via plasmid transfection. Successful overexpression of Prox1 was confirmed by qPCR (Fig. 7A, P < 0.0001, n = 3, One-Way ANOVA). Notably, MBP protein levels were significantly higher in the Prox1 overexpression group compared to the empty vector group (Fig. 7B, C, P < 0.0001, n = 3, One-Way ANOVA), suggesting that Prox1 may play a role in enhancing MBP synthesis.

## 4 Discussion

PNI is common in clinical practice and often leads to severe functional impairments, including damage to connective tissue, myelin sheaths, and axons (Zhang et al., 2021, Dun and Parkinson, 2018), which can result in permanent disabilities and significantly impact quality of life. The sciatic nerve, responsible for motor control and sensory input in the lower limbs, is frequently injured, causing motor loss and pain. Despite advances in diagnostics, microsurgery, and treatments like Puerarin (Wu et al., 2014),and inhibitor of DNA binding2 (Huang et al., 2021), functional recovery remains limited. Improper or delayed treatment can lead to irreversible nerve damage, making peripheral nerve repair and regeneration crucial areas of research. It has been reported that 92 out of 459 proteins were differentially expressed in the sciatic nerve post-injury (Msheik et al., 2023). The SNI model is commonly used for evaluating mechanistic studies of peripheral nerve repair (Manzanera Esteve et al., 2023, An et al., 2022). Thus, a mouse model of SNI was used in our study.

In a rat model of PNI, myelin sheath damage impairs nerve regeneration (Sundaram et al., 2023, Yuan et al., 2021, Wang et al., 2018). Although myelin structure differs slightly between the central and peripheral nervous systems, both involve axons encased by glial cells in concentric layers. Myelin damage can lead to neurological disorders like Alzheimer's disease (McMurran et al., 2016), multiple sclerosis (Matute, 1998), and pathological neuropathic pain (Noorani et al., 2019). Myelin damage is commonly observed in various PNI (Shin et al., 2023), and the great plasticity of Schwann cells (SCs) and myelinating glia of the peripheral nervous system are critical for peripheral nerve regeneration. SCs provide support to myelin regeneration in peripheral nervous system by secreting a range of neurotrophic factors to promote the growth of nerve fibers (Nomura et al., 2006). In rat spinal cord injury models, SCs transplantation reduces cyst size, provides neuroprotection, and promotes axonal regeneration (Khan et al., 2022, Assinck et al., 2017). Due to their established repair capabilities, SCs represent promising candidates for transplantation therapies in both spinal cord injuries and PNI. MBP, synthesized by oligodendrocytes in the CNS and SCs in the PNS, is key to peripheral nerve repair (Bolino, 2021, Bosch-Queralt et al., 2023, Nocera and Jacob, 2020). MBP expression correlates with myelin regeneration and functional recovery after SNI (Liu et al., 2019, Lee et al., 2022, Ji et al., 2023), therefore, serum MBP level and myelin content are both used for nerve injury assessment clinically (Okura et al., 2022, Balaji et al., 2022, Oh et al., 2019). Our study observed significant myelin damage in the sciatic nerve at Day 7 post-injury, including folding, disarray, and even demyelination, and with signs of regeneration by Day 14 with EM, H&E, and luxol fast blue staining. *This indicates the sciatic nerve damage on mice was most obvious on Day 7 post-injury, with signs of regeneration beginning on Day 14 post-injury.*

In our study, control mice showed well-organized axons and intact myelin sheaths. In the injured sciatic nerve model, axonal damage was observed from Day 4 post-injury, characterized by vacuoles and increased spacing. Axonal spacing continued to increase with disorganization by Days 5 and 6. By Day 7, myelin sheaths were sparse and disordered. On Day 14, axons were more orderly, myelin fibers increased, and axonal shrinkage reduced. EM analysis confirmed the most severe myelin damage on Day 7, with recovery by Day 14. IHC showed a decline in MBP levels starting Day 3, reaching a minimum on Day 7, and returning to normal by Day 14. *All of these suggest the positive correlation between extend of myelin sheath regeneration and MBP expression level post PNI, which is consistent to the previous founding that MBP expression was closely associated with functional recovery from SNI (*Liu et al., 2019*).*

Injured myelin sheaths and neurons trigger secondary processes like inflammation and fibrosis, which impair regeneration (Yuan et al., 2022, Ji et al., 2023, Ahuja et al., 2017, Schwab and Bartholdi, 1996). Myelin debris also inhibit axonal and myelin regeneration (Chen et al., 2000, Boghdadi et al., 2018, Kotter et al., 2006). We hypothesize that by Day 3 post-SNI, damaged myelin accumulates, hindering repair, with the most severe damage and lowest MBP levels seen on Day 7. However, the reasons for MBP recovery and myelin repair by Day 14 remain unclear.

Prox1 is also a key regulator of venous endothelial cell differentiation into lymphatic vessels, promoting edema resolution and nerve recovery (Wigle et al., 2002). In consistent, our previous research also confirmed that new lymphatic vessels in the peripheral nervous system reduce tissue edema by clearing excess matrix, inflammatory cells, and myelin debris, thereby promoting myelin sheath formation and sciatic nerve recovery (Meng et al., 2020). Additionally, Prox1 levels of SNI mouse significantly increased by Day 7 post-injury, suggesting its potential role in nerve repair and regeneration (Meng et al., 2020). However, the dynamic changes in Prox1 expression during recovery and its exact role in myelin regeneration are yet to be fully explored.

In the central nervous system, Prox1 is crucial for oligodendrocyte precursor cell survival, proliferation, and central nervous system regeneration (Franklin and Ffrench-Constant, 2008). Prox1 has been documented to promote oligodendrocyte differentiation and facilitates myelin sheath regeneration in a mouse spinal cord model (Kato et al., 2015). During this process, Prox1 expression increases to enhances differentiation and MBP level, while its knockdown impairs differentiation, reduces MBP, and promotes apoptosis (Chang and Teng, 2018). However, its role in the peripheral nervous system and myelin regeneration is unclear. We observed co-localization of MBP and Prox1 proteins in injured sciatic nerves, with Prox1 levels increasing at Day 3, peaking at Day 7, and decreasing by Day 14 post-injury. Given that myelin regeneration correlates with MBP levels, we infer that Prox1 promotes myelin sheath regeneration in the peripheral nervous system. To validate this, we overexpressed PROX1 in Schwann cells and observed an increase of MBP protein level. Our in vitro experiments further suggest that following sciatic nerve myelin injury, Prox1 expression begins to increase to promote myelin regeneration and remodeling by inducing MBP expression. By studying SNI and the changes in Prox1/MBP expression, along with increased MBP levels following PROX1 overexpression in vitro, we identified Prox1's potential role in promoting peripheral nerve regeneration, particularly in myelin sheath repair for the first time. We also characterized the morphological changes and recovery timeline of myelin sheaths post-SNI for the first time.

While our study provides evidence that Prox1 enhances myelin sheath regeneration in the SNI, a critical test of its direct functional role would require conditional knockout or knockdown of Prox1 in vivo. Such experiments are essential to determine whether Prox1 is necessary for myelination initiation, progression, or maintenance in a physiological context, independent of compensatory mechanisms that may arise in vitro. Although technical constraints—particularly the challenge of achieving efficient Schwann cell-specific Prox1 deletion in mature nerves—precluded direct in vivo functional validation in this study, future work employing inducible, cell-type-specific Cre lines (*Mpz-CreERT2*) for postnatal Prox1 ablation, or AAV-delivered CRISPR-based epigenome editing, should establish whether the in vitro findings translate to the intact nervous system.

## Author statement

Linlin Jie drafted the manuscript. Linlin Jie and Fanwei Meng designed experiments, collected data and interpreted results. The experiments were carried out by Linlin Jie and Jun Ren. Jie Liu proofread the article draft. Xuening Jing performed data analysis. Xiaoni Kong contributed to the methodology and performed the Western blot experiments. Jun Wu and Fanwei Meng co-supervised the project and contributed to discussion and conclusion presented in the manuscript. All authors engaged in discussions regarding the results and contributed to the final version of the manuscript.

## CRediT authorship contribution statement

**Jun Wu:** Supervision, Project administration, Conceptualization. **Xiaoni Kong:** Methodology. **Fanwei Meng:** Writing – original draft, Supervision, Project administration, Funding acquisition, Conceptualization. **Xuening Jing:** Formal analysis, Data curation. **jie linlin:** Writing – original draft, Validation, Software, Methodology, Investigation. **Jun Ren:** Software, Methodology. **Jie Liu:** Writing – review & editing.

## Ethical approval

All animal experiments involved in this study were approved by the Ethics Committee of the Shandong College of Traditional Chinese Medicine, China and the Approval Number is 2022–02.

## Funding

This study was funded by the Medical and Health Science and Technology Development Plan Project of Shandong Province (No. 202101011003), Medical and Health Science and Technology Development Plan Project of Shandong Province ((No. 202205010845), the Joint Project by National Division and Provincial Bureau of TCM (GZY-KJS-SD-2023–052) and Yantai School-Government Integration Development Project (2023XDRHXMXK08). We acknowledge the contributions of these funding sources in facilitating our research and the support from Yantai City Key Laboratory for the Prevention and Treatment of Metabolic Diseases with Traditional Chinese Medicine (In Preparation)

## Declaration of Competing Interest

The authors declare that we have no known competing financial interests or personal relationships that could have appeared to influence the work reported in this paper.

## Data Availability

The data generated in this study can be provided by the corresponding author on request.
